# Evolution of National Guidelines on Medicines Used to Treat COVID-19 in Pregnancy in 2020–2022: A Scoping Review

**DOI:** 10.3390/jcm12134519

**Published:** 2023-07-06

**Authors:** Emeline Maisonneuve, Odette de Bruin, Guillaume Favre, Anna Goncé, Serena Donati, Hilde Engjom, Eimir Hurley, Nouf Al-Fadel, Satu Siiskonen, Kitty Bloemenkamp, Hedvig Nordeng, Miriam Sturkenboom, David Baud, Alice Panchaud

**Affiliations:** 1 Institute of Primary Health Care (BIHAM), University of Bern, 3012 Bern, Switzerland; alice.panchaudmonnat@unibe.ch; 2 Graduate School for Health Sciences (GHS), University of Bern, 3012 Bern, Switzerland; 3 Materno-Fetal and Obstetrics Research Unit, Woman-Mother-Child Department, Lausanne University Hospital, 1011 Lausanne, Switzerland; guillaume.favre@chuv.ch (G.F.); david.baud@chuv.ch (D.B.); 4 Department of Obstetrics, WKZ Birthcentre, Division Woman and Baby, UMC Utrecht, 3584 CX Utrecht, The Netherlands; o.debruin-3@umcutrecht.nl (O.d.B.); k.w.m.bloemenkamp@umcutrecht.nl (K.B.); 5 Julius Global Health, University Medical Center Utrecht, 3584 CX Utrecht, The Netherlands; 6 BCNatal—Fetal Medicine Research Center, Hospital Clínic and Hospital Sant Joan de Déu, Universitat de Barcelona, 08028 Barcelona, Spain; agonce@clinic.cat; 7 Institut d’Investigacions Biomèdiques August Pi i Sunyer (IDIBAPS), 08036 Barcelona, Spain; 8 National Centre for Disease Prevention and Health Promotion, Istituto Superiore di Sanità, 00161 Rome, Italy; serena.donati@iss.it; 9 Department of Health Promotion, Department of Health Registry Research and Development, Norwegian Institute of Public Health, N-5808 Bergen, Norway; hildemarie.engjom@fhi.no; 10 Pharmacoepidemiology and Drug Safety Research Group, Department of Pharmacy and PharmaTox Strategic Initiative, Faculty of Mathematics and Natural Sciences, University of Oslo, 0316 Oslo, Norway; eimirhurley@gmail.com (E.H.); h.m.e.nordeng@farmasi.uio.no (H.N.); 11 Saudi Food and Drug Authority, Riyadh 13513-7148, Saudi Arabia; nsfadel@sfda.gov.sa; 12 Division of Pharmacoepidemiology & Clinical Pharmacology, Utrecht Institute for Pharmaceutical Sciences, Utrecht University, 3584 CG Utrecht, The Netherlands; s.j.siiskonen@uu.nl (S.S.); m.c.j.sturkenboom@umcutrecht.nl (M.S.); 13 Service of Pharmacy, Lausanne University Hospital, University of Lausanne, 1011 Lausanne, Switzerland

**Keywords:** COVID-19, national guidelines, clinical practice guidelines, pregnancy

## Abstract

The lack of inclusion of pregnant women in clinical trials evaluating the effectiveness of medicines to treat COVID-19 has made it difficult to establish evidence-based treatment guidelines for pregnant women. Our aim was to provide a review of the evolution and updates of the national guidelines on medicines used in pregnant women with COVID-19 published by the obstetrician and gynecologists’ societies in thirteen countries in 2020–2022. Based on the results of the RECOVERY (Randomized Evaluation of COVID-19 Therapy) trial, the national societies successively recommended against prescribing hydroxychloroquine, lopinavir–ritonavir and azithromycin. Guidelines for remdesivir differed completely between countries, from compassionate or conditional use to recommendation against. Nirmatrelvir–ritonavir was authorized in Australia and the UK only in research settings and was no longer recommended in the UK at the end of 2022. After initial reluctance to use corticosteroids, the results of the RECOVERY trial have enabled the recommendation of dexamethasone in case of severe COVID-19 since mid-2020. Some societies recommended prescribing tocilizumab to pregnant patients with hypoxia and systemic inflammation from June 2021. Anti-SARS-CoV-2 monoclonal antibodies were authorized at the end of 2021 with conditional use in some countries, and then no longer recommended in Belgium and the USA at the end of 2022. The gradual convergence of the recommendations, although delayed compared to the general population, highlights the importance of the inclusion of pregnant women in clinical trials and of international collaboration to improve the pharmacological treatment of pregnant women with COVID-19.

## 1. Introduction

The sudden onset of the coronavirus disease 2019 (COVID-19) pandemic has challenged the existing approaches to evaluate clinical evidence and generate Clinical Practice Guidelines (CPGs). Early in the pandemic, physicians lacked evidence-based guidelines to treat COVID-19 patients. The first treatment strategies were generated from anecdotal observations, in vitro assays, and hypothetical pathophysiological mechanisms, with large differences from one hospital to another. The RECOVERY (Randomized Evaluation of COVID-19 therapy), REMAP-CAP (Randomised, Embedded, Multi-factorial, Adaptive Platform Trial for Community-Acquired Pneumonia), and WHO-SOLIDARITY clinical trials were based on extensive multicenter collaboration, adaptive design in randomized controlled trials (RCTs) and the utilization of preprints. They provided an influx of good-quality evidence and led to profound clinical practice changes over time for patients who were not pregnant [1,2,3,4,5,6,7,8,9]. However, it was particularly challenging to derive evidence regarding treatment guidelines for populations with additional treatment safety concerns, such as the pediatric and pregnant patients. As a matter of fact, pregnant women were not included in most RCTs evaluating the effectiveness of treatments used in COVID-19, even though experiences from previous outbreaks and pandemics have highlighted the priority for knowledge about access to treatment for pregnant women [10,11,12]. Moreover, finding the right treatment would have been particularly relevant in the obstetrics population, since pregnant women have been identified early on as significantly more likely than nonpregnant women of the same age to be admitted to an intensive care unit, receive invasive ventilation, and die [13,14]. Later on, COVID-19 in pregnancy was also associated with a higher risk of preterm delivery and a higher risk of infant admission in neonatal intensive care unit [14,15]. National guidelines for medications used to treat SARS-CoV-2 infection during pregnancy were derived from the evolving and growing literature and from treatment guidelines for nonpregnant adults with COVID-19 and have changed significantly over time. Our aim was to provide a scoping review of the evolution of the national guidelines on medicines used to treat COVID-19 in pregnant women from March 2020 to December 2022. Our research does not intend to provide the current guidelines on pharmacological treatments, but rather to compare and highlight the discrepancies between the different national recommendations, especially in the early stages of the pandemic.

## 2. Materials and Methods

### 2.1. Selection Criteria

We focused on the national CPGs from nine countries participating in the CONSIGN (COVID-19 infectiON and medicineS In preGNancy) working group (Belgium, Canada, France, Italy, Norway, Saudi Arabia, Spain, Switzerland, The Netherlands), as well as four countries whose national guidelines are written in English (Australia, India, UK, and USA) and those issued by the World Health Organization (WHO). The period of analysis was from 1 March 2020 to 31 December 2022. Regional CPGs and reviews of existing guidelines were excluded.

### 2.2. Documents of Interest and Research Process

**Figure 1:**
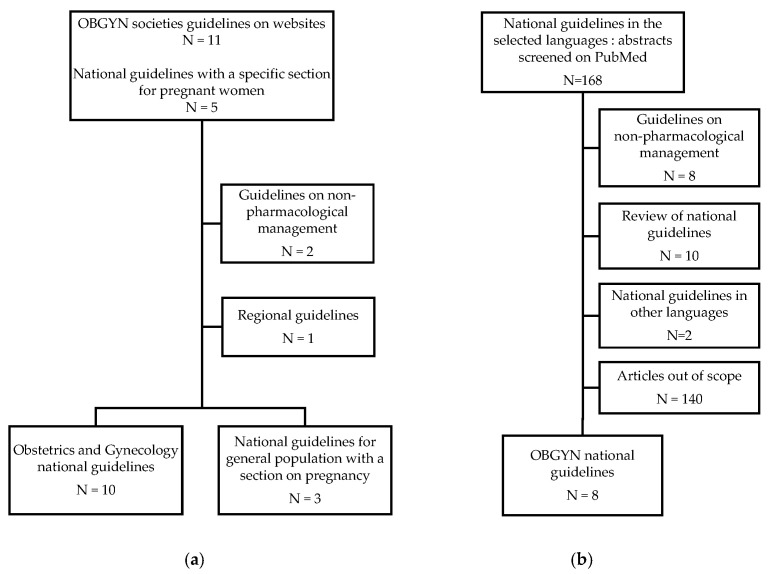
Selection process of national guidelines on COVID-19 medicines during pregnancy: (**a**) among official websites of the national societies in obstetrics and gynecology; (**b**) among articles on national guidelines on PubMed. OBGYNs: Obstetricians and Gynecologists.

CPGs are statements that include recommendations intended to optimize patient care. Rapid Guidelines are defined as CPGs which are completed in a 1-to-3 month timeframe and are alternatives to comprehensive CPGs to provide guidance in response to an emergency [16]. First, we searched for the CPGs published by Obstetrician Gynecologist (OBGYN) national societies on their official websites. If there was no OBGYN national guideline, national guidelines for the general population were considered only if they were providing specific recommendation for pregnancy. Finally, all other national guidelines including a focus on pregnant women, published by national medicines agencies, professional societies, government organizations, such as health ministries’ websites, were considered (Table 1). To complete the research process, the literature search was conducted on the MEDLINE (PubMed) database by E.M. and O.d.B. to identify all relevant CPGs published between March 2020 and December 2022. Combinations of the following terms were used: “COVID-19”, “SARS-CoV-2”, “treatment”, “medicines”, “medication”, “drug”, “pharmacologic”, “pregnancy”, “pregnant”, “guidelines”, “recommendations”. Articles and guidelines from these searches and relevant references cited in those articles were reviewed (Figure 1). National guidelines from Australia, Canada, India, Saudi Arabia, UK, and the USA were in English [17,18,19,20,21,22]. Belgian guidelines for the general population with a focus on pregnancy were displayed in national languages and English; Italian guidelines for the general population represented a reference for drug prescription during pregnancy taking into account the content of the summary product characteristics’ indication and the benefit/risk profile of individual cases; and Dutch guidelines for pregnant women were available in Dutch and English [21,23,24]. National guidelines in other languages were translated by the members of the CONSIGN working group (O.d.B. translated the Belgian and Dutch national online guidelines; E.M. translated guidelines from France and Switzerland; and S.D., H.E. and A.G. translated the Italian, Norwegian and Spanish guidelines, respectively) [21,23,25,26,27,28,29]. Articles displaying national guidelines published in English and French on PubMed as of 5 April 2023 were also included.

### 2.3. Outcomes of Interest

This review focuses on the evolution of the national guidelines on medicines recommended to treat COVID-19 in pregnancy from 2020 to 2022, but not on other medication administered routinely in pregnancy, nor vaccination, or current treatment guidelines, as these topics have already been the subject of systematic reviews [30,31]. The standard of care of COVID-19 (e.g., hydration, antipyretics in case of fever, and respiratory support) is of utmost importance, but outside the scope of this review.

### 2.4. Recommendation Grading

In order to harmonize all terms used in the national guidelines, we used the same terminology and corresponding color codes for all countries as published in the WHO guidelines and the Australian guidelines for Australia, as they are very similar [32]. These recommendations’ grading and corresponding color codes and definitions are summarized in Table 1 [33]. In addition, the categories “consider” (or “no absolute contraindication” or “can be offered”) and “compassionate use” were used to accurately represent the guidelines. “Compassionate use” (also called “expanded access”) is the use of an unapproved drug under special forms of new investigational drug applications outside of a clinical trial by people with serious or life-threatening conditions who do not meet the enrollment criteria for the clinical trial in progress.

This study was reported according to the Preferred Recorded item for Systematic reviews and meta-analyses extension for Scoping reviews (PRISMA-ScR) checklist.

## 3. Results

Based on our search on official websites and scientific literature, we found OBGYNs national expert society guidelines available for Australia, Canada, France, India, Spain, Switzerland, The Netherlands, UK and the USA. In Norway, clinical guidelines were issued by the Norwegian Society of Gynecology and Obstetrics in collaboration with the Norwegian Institute of Public Health [29].

In the USA, additionally to the OBGYN national expert guidelines, the guidelines on treatment came mostly from the National Institutes for Health (NIH), which is a part of the USA department of Health and Human services, and the largest biomedical research agency in the world; NIH’s guidelines concern the whole population including specific parts for pregnant women. Saudi Arabian guidelines were issued by the Saudi Society of Fetal Maternal medicine and the Ministry of Health [17,34].

OBGYN national expert societies’ guidelines regarding COVID-19 treatment were not available for Belgium and Italy, and recommendations were drawn from national medicine agency guidelines. Thus, in Belgium, the Federal Agency for Medicines and Health Products (AFMPS) guidelines specific to pregnancy advised referring to international recommendations, such as WHO, Royal College of Obstetricians and Gynaecologists (RCOG) from the UK, and NIH guidelines. In Italy, obstetricians used the Italian Medicines Agency (AIFA) recommendations, as well as RCOG and WHO interim guidelines.

International recommendations were based on WHO guidelines as the International Federation of Gynecology and Obstetrics (FIGO) referred to the national guidelines of FIGO’s national members societies, to WHO guidelines, and to Center for Disease Control and Prevention (CDC) recommendations (based on NIH guidelines for treatments).

During the acute period of the pandemic, the national online guidelines were updated frequently; the NIH recommendations were updated 62 times in three years and included a specific section for pregnant women (Table 2) [35]. The different medications which have been considered to treat COVID-19 in pregnant women in the national guidelines between 2020 and 2022 are described by group of medicines in following paragraphs.

### 3.1. Antibiotics

Several antibiotics were considered empirically effective treatments in the early months of the pandemic, before the large trials results showed otherwise. They were used as a first-line treatment against COVID-19 from the very beginning, due to past successful performance in other viral epidemics, as well as to treat pulmonary co-infections [42]. Since mid-2020, most national guidelines did not recommend prescribing antibiotics such as amoxycillin, unless an additional bacterial pulmonary infection was suspected [18,21,25,26,27,39]. Azithromycin was initially considered to treat COVID-19, since this macrolide antibiotic has not only antimicrobial properties, but also immunomodulatory activity [43]. Regarding its safety in pregnancy, most studies found no increase in major congenital malformations after azithromycin exposure during the first trimester [44]. In February 2021, the RECOVERY collaborative group published their results indicating that azithromycin alone (without hydroxychloroquine) did not improve survival or other clinical outcomes when compared to the usual standard of care [4]. Thus, the national guidelines successively recommended not to prescribe azithromycin (July 2021 in the USA and August 2021 in the UK), unless an additional bacterial pulmonary infection was suspected [40,45].

### 3.2. Antimalarials

Chloroquine and hydroxychloroquine are oral drugs used for the treatment of malaria and some autoimmune conditions, respectively, and both drugs have in vitro activity against SARS-CoV-2. Hydroxychloroquine is considered safe during pregnancy with no ophthalmological repercussions in the infant observed so far [46]. At the start of the pandemic, hydroxychloroquine was recommended in India and Saudi Arabia [34,36]. It was “under assessment” and could be prescribed in pregnant women only within a context of a clinical trial in Australia, France, Italy, Norway and The Netherlands until May–June 2020 [22,26,29,47,48]. In Spain, the national guidelines did not mention hydroxychloroquine at the same period [27]. However, in May 2020, Spain launched a randomized double-blinded, placebo-controlled multicenter clinical trial for pregnant women, aiming to assess the safety and efficacy of hydroxychloroquine to reduce maternal viral shedding, prevent incident SARS-CoV-2 infection, and severe disease in pregnant women. This trial enrolled 129 patients, but the results have not been published yet [49]. In contrast, other countries such as Australia, Belgium, and the USA never recommended hydroxychloroquine for pregnant women [17,50,51]. According to the results of the RECOVERY trial, published in June 2020, hydroxychloroquine was ineffective and should not be prescribed [3]. Thus, the WHO and many national societies of OBGYNs have successively recommended against prescribing this drug from December 2020 onwards [29,33,40,45,47].

### 3.3. Antivirals

- Remdesivir

Remdesivir is a novel broad antiviral nucleotide pro-drug which was shown to inhibit the replication of SARS coronaviruses in vitro. Initial studies suggested some benefit in shortening the median recovery time, but it has not shown any benefit in reducing mortality [52]. Results from the compassionate-use program provide strong support that remdesivir has a favorable risk–benefit balance with its high rates of clinical recovery [53]. The conditions of use of remdesivir in pregnancy differed from country to country. It was possible to prescribe remdesivir since Spring 2020 in Italy and in Norway, after individual evaluation by an infectious disease specialist (only in a clinical trial setting in Norway from November 2020) [21,29]. It was considered for compassionate use in deteriorating pregnant patients with COVID-19, from 2020 in the USA and from 2021 in the UK [20,45]. On the contrary, Dutch recommendations suggested avoiding remdesivir because the potential effectiveness was limited and a safety profile during pregnancy was insufficiently characterized [54].

- Lopinavir–ritonavir

Lopinavir–ritonavir is a combination of protease inhibitors that are currently approved and have been largely used for the treatment of human immunodeficiency virus infection, and it demonstrated in vitro activity against coronaviruses. Between April 2020 and August 2021, Belgian, Saudi Arabian, Dutch, UK, and USA guidelines successively recommended not to prescribe the combination lopinavir–ritonavir against COVID-19 [24,34,40,45,54]. In Norway, infectious disease specialists were reluctant to recommend its use outside a clinical trial setting [29].

- Nirmatrelvir–ritonavir

Nirmatrelvir is a peptidomimetic inhibitor of the coronavirus 3C-like (3CL) protease which prevents viral replication. In December 2021, the USA Food and Drug Administration (FDA) issued an emergency use authorization for nirmatrelvir–ritonavir for the treatment of mild-to-moderate COVID-19 in adults and some pediatric patients who are at high risk for progression to severe COVID-19. In response, the Society for Maternal Fetal Medicine (SMFM) supported the use of nirmatrelvir–ritonavir for the treatment of pregnant patients with COVID-19 who met clinical qualifications [55]. In March 2022, the RCOG recommended that pregnant women should be offered the opportunity to enroll, if they were eligible, in clinical trials. Then, in December 2022, the RCOG contraindicated nirmatrelvir–ritonavir, following advice from the Medicines and Healthcare Regulatory Agency of the UK [39]. The main and specific safety signals of nirmatrelvir are disease recurrence, dysgeusia, abdominal pain and skin toxicity [56].

### 3.4. Corticosteroids

Initial concerns that corticosteroids could reduce viral clearance and increase the severity of infection resulted in reluctance to prescribe corticosteroids to pregnant women, even for established indications such as fetal lung maturation [57,58,59]. Thus, in March 2020, most guidelines called for caution before the administration of corticosteroids to COVID-19-positive pregnant women, especially in the context of critically ill patients, and they could only be prescribed after a multidisciplinary discussion with specialists in maternal–fetal medicine and infectious diseases [57,59]. In June 2020, the RECOVERY trial demonstrated that the use of corticosteroids significantly reduced the mortality within 28 days (age-adjusted rate ratio, 0.83; 95% confidence interval (CI), 0.75 to 0.93; *p* < 0.001) in patients who required mechanical ventilation or oxygen supplementation [2]. The regimen studied in the RECOVERY trial was dexamethasone 6 mg (orally or intravenously) for up to 10 days. The prolonged use of corticosteroids has been associated with intrauterine growth retardation and low birth weight in children of mothers receiving long-term corticosteroid treatment for chronic conditions such as lupus, asthma and organ transplants. The specific role of these pathologies cannot be ruled out. On the mechanistic level, fluorinated corticosteroids that readily cross the placenta like dexamethasone might have a higher risk to impact the fetus than the nonfluorinated ones which have a higher metabolizing rate by the placenta [60]. Although corticosteroids were also recommended for pregnant women with severe COVID-19 by the national OBGYN societies after the RECOVERY trial results, there was ongoing debate about the choice of fluorinated or nonfluorinated corticosteroid given different propensity to cross the placenta. Therefore, the societies of Dutch and Saudi Arabian OBGYNs and the RCOG have recommended using nonfluorinated corticosteroids for maternal severe COVID-19 to avoid fetal exposure [23,34,45]. The different regimens are shown in Table 3 and its legend.

### 3.5. Tocilizumab

Tocilizumab is a humanized monoclonal IgG1 antibody which binds and inhibits both soluble and membrane-bound interleukine-6 (IL-6) receptors. This inhibits the pro-inflammatory activity of IL-6, which may be increased in patients with COVID-19. Anti-IL-6 therapy is usually used as an immunosuppressant in rheumatoid arthritis and its use requires monitoring of liver function [46]. Transplacental transport of IgG is thought to be very low during the first trimester of pregnancy. There is limited information on the safety of tocilizumab during the second and third trimesters. In April 2021, the adaptive randomized controlled REMAP-CAP trial demonstrated an absolute reduction in mortality of 8.5% when it was administered within 24 h of admission to critical care [7]. In May 2021, the RECOVERY trial found an absolute mortality reduction of 4% when it was given to patients with hypoxia (oxygen saturation < 92% on air or requiring oxygen therapy) and evidence of systemic inflammation (C-reactive protein [CRP] ≥ 75 mg/L) [5]. Improvements in mortality rates have been confirmed in subsequent meta-analyses [61]. With regard to the national guidelines, the RCOG asked to strongly consider tocilizumab in August 2021 and recommended to give tocilizumab (or sarilumab if not available) if needing escalation of care and/or CRP > 75 mg/L in December 2021 [62]. The potential use of tocilizumab was also mentioned in the Norwegian guidelines from May 2021 [29]. Since December 2021, the Dutch guidelines have recommended tocilizumab as the first-choice drug for the treatment of COVID-19-positive pregnant women if they qualify for anti-IL-6 therapy; the indication and dosage of tocilizumab should ideally be determined by a multidisciplinary team including at least a pulmonologist and/or internist and/or intensivist and a perinatologist [54]. There was a conditional recommendation for tocilizumab also in Australia and India [32,36]. On the contrary, the Belgian guidelines from April 2020 recommended that it should not be prescribed to pregnant women unless clearly necessary [63]. Given the paucity of data, the NIH recommended against the use of tocilizumab during pregnancy from October 2021 [40,64].

### 3.6. Anti-SARS-CoV-2 Monoclonal Antibodies

Anti-spike monoclonal antibodies (mABs) received emergency use authorization from the USA FDA for the outpatient treatment of mild-to-moderate COVID-19 in patients at increased risk of severe outcomes. Although this treatment does not seem to have been associated with an increase in adverse pregnancy outcomes, more data and longer follow-up are needed to better characterize its safety profile in pregnancy [65].

In May 2021, the USA FDA expanded the emergency use authorization to include pregnancy as a qualifying condition for monoclonal antibodies [35]. From October 2021, the use of anti-SARS-CoV-2 mABs has been considered for pregnant women with COVID-19 in the US, especially those with additional risk factors for severe disease. In January 2022 in Switzerland, monoclonal antibodies could be offered in COVID-19 during pregnancy after a detailed multidisciplinary counselling about the benefits and risks. At that time the Omicron variant was the one mostly circulating and casirivimab/imdevimab was not efficient on this variant. Unless other variants were identified by individual testing, sotrovimab was the only mAB recommended based on the following prescription criteria: mild or moderate infection AND a duration of symptoms of ≤5 days AND co-morbidities AND gestational age > 12 weeks of gestation AND not fully vaccinated status (i.e., two doses) [66]. In 2022, the RCOG stated that the decision about which preparation to offer might depend on the variant and asked to refer to the most up-to-date information from their Medicine and healthcare products regulatory agency [39]. Anti-SARS-CoV-2 mABs were no longer recommended by the Dutch, NIH and WHO guidelines at the end of 2022, because the predominant Omicron subvariants were not susceptible [64].

### 3.7. Convalescent Plasma

In August 2020, the USA FDA gave emergency use authorization for convalescent plasma therapy to treat COVID-19 using donor plasma from people with high titers of anti-SARS-CoV-2 antibody. In December 2021, the USA FDA restricted its authorization to the treatment of COVID-19 in patients with immunosuppressive disease or receiving immunosuppressive treatment. In 2021, The Netherlands and the UK recommended not to prescribe convalescent plasma to pregnant women, except in the context of clinical trials [54]. In Norway, it could be prescribed after individual evaluation by an infectious disease specialist [29]. The other OBGYN national guidelines did not mention convalescent plasma to treat COVID-19. Convalescent plasma administered to pregnant women with severe COVID-19 has not been associated with adverse pregnancy outcomes. However, the current evidence relies on small case reports or case series and thus more studies are required to characterize the safety profile of this treatment [67].

### 3.8. Intravenous Immunoglobulins

Polyvalent intravenous immunoglobulins (IVIg) are commonly used in pregnancy for other indications, such as autoimmune thrombocytopenia, and have an acceptable safety profile [68]. The most frequent side effect is headache. In The Netherlands, prescription was not recommended except in a clinical trial setting. Since mid-2020, the NIH and Saudi Arabian guidelines have recommended against the use of polyvalent IVIg to treat COVID-19 in adults, except in a clinical trial setting [34,40]. In Norway, it could be prescribed after individual evaluation by an infectious disease specialist [29]. Otherwise, national guidelines did not mention anything about polyvalent IVIg in pregnant women with COVID-19.

### 3.9. Thromboprophylactic Agents

Reports of pulmonary microthrombi on post-mortem examination in COVID-19 patients and case series reporting a higher incidence of venous thromboembolism (VTE) steered the use of anticoagulation in patients with COVID-19 [69]. Since the early pandemic, most national guidelines recommended prophylactic anticoagulation conditional on hospitalization and/or personal thromboembolic risk factors in pregnancy. In Belgium, thromboprophylaxis with low-molecular-weight heparin (LMWH) was recommended for women with COVID-19 either hospitalized and symptomatic or nonhospitalized with severe symptoms and immobilization [70]. In Spain, LMWH was recommended for all women with COVID-19 irrespective of symptoms and hospitalization [27]. Prophylactic anticoagulation was conditional on hospitalization in Norwegian, Swiss, UK and USA guidelines, and on individual risks factors for VTE in Australian and French guidelines [26,28,32,39,64]. The VTE treatment should continue for the duration of inpatient stay and for at least 10 days after discharge in Norway, Switzerland and the UK [19,28,37,62].

However, as severe COVID-19 can be associated with thrombocytopenia, the RCOG recommended stopping thromboprophylaxis, and seek hematology advice if an infected pregnant woman has a platelet count below 50 × 10^9^/L [19]. Heparin does not cross the placenta and is widely used in pregnancy.

The timeline of the evolution of the national guidelines for pregnant women from March 2020 to December 2022 is presented by medicine group in Table 3, associated with the corresponding conditions of use (i.e., clinical trial only, compassionate use).

**Table 3:** Evolution of national guidelines on treatments in pregnant women with COVID-19: timeline from March 2020 to December 2022 in 13 countries and the World health Organization, displayed by medicine group.

Medicine	Year	2020										2021				2022				Ref
	Country	March	April	May	June	July	August	September	October	November	December	Q1	Q2	Q3	Q4	Q1	Q2	Q3	Q4	
Azithromycin	Australia																			[32]
	Belgium																			[71]
	Canada																			
	France																			[26]
	India																			[72]
	Italy			§																[21]
	Norway																			[37]
	S. Arabia																			
	Spain																			
	Switzerland																			
	The Netherlands																			[38]
	UK																			[45]
	USA																			[40]
	WHO																			
Hydroxychloroquine	Australia																			[32]
	Belgium																			[73]
	Canada																			
	France																			[26]
	India																			[36]
	Italy																			[47]
	Norway																			[37]
	S. Arabia																			[34]
	Spain																			
	Switzerland																			
	The Netherlands																			[38]
	UK																			[45]
	USA																			[74]
	WHO																			[75]
Remdesivir	Australia																			[32]
	Belgium																			[76]
	Canada																			
	France																			[26]
	India																			[72]
	Italy																			[21]
	Norway									†										[37]
	S. Arabia																			[17]
	Spain																			
	Switzerland																			
	The Netherlands					††														[38]
	UK																			[45]
	USA																			[74]
	WHO																	†††		[77]
Lopinavir-Ritonavir	Australia																			[32]
	Belgium																			[76]
	Canada																			
	France																			[26]
	India																			[36]
	Italy																			[21]
	Norway									†										[37]
	S. Arabia																			[17]
	Spain																			
	Switzerland																			
	The Netherlands																			[38]
	UK																			[45]
	USA																			[64]
	WHO																			[75]
Nirmatrelvir-Ritonavir	Australia																			[32]
	Belgium																			[78]
	Canada																			
	France																			
	India																			
	Italy																			[21]
	Norway																			[37]
	S. Arabia																			
	Spain																			
	Switzerland																			
	The Netherlands																			[38]
	UK																			[39]
	USA																			[64]
	WHO																			[75]
Corticosteroids (systemic)	Australia							*												[32]
	Belgium							* αβ	χ											[79]
	Canada												*							[80]
	France								*											[26]
	India												* αβ	χ						[72]
	Italy								* αβ	φ										[21]
	Norway									*						λ				[37]
	S. Arabia						* δε													[17]
	Spain			* γ																[27]
	Switzerland																			
	The Netherlands										*		ζ							[38]
	UK		* δε																	[81]
	USA		η											*						[64]
	WHO							*								δεζ				[75]
Tocilizumab	Australia																			[32]
	Belgium																			[79]
	Canada																			
	France																			[26]
	India																			[72]
	Italy																			[21]
	Norway															†				[37]
	S. Arabia																			
	Spain																			
	Switzerland																			
	The Netherlands																		±	[38]
	UK																			[45]
	USA																			[64]
	WHO																			[77]
Anti-IL 1	Australia																			[32]
	Belgium																			
	Canada																			
	France																			
	India																			
	Italy																			[21]
	Norway																			
	S. Arabia																			
	Spain																			
	Switzerland																			
	The Netherlands																			[38]
	UK																			
	USA																			[64]
	WHO																			
Anti-SARS-CoV-2 monoclonal antibodies	Australia														Σ		$			[32]
	Belgium																			[79]
	Canada																			
	France																			[26]
	India																			[72]
	Italy																≈			[21]
	Norway															†				[37]
	S. Arabia																			
	Spain																			
	Switzerland															Σ				[66]
	The Netherlands																			[38]
	UK															∞			∞	[45]
	USA																			[64]
	WHO																			[77]
Convalescent plasma	Australia																			[32]
	Belgium																			
	Canada																			
	France																			[26]
	India																			[72]
	Italy																			
	Norway																			[37]
	S. Arabia																			[17]
	Spain																			
	Switzerland																			
	The Netherlands																			[38]
	UK																			[45]
	USA																		√	[64]
	WHO																			[77]
IVIg	Australia																			[32]
	Belgium																			
	Canada																			
	France																			
	India																			
	Italy																			
	Norway																			[37]
	S. Arabia																			[34]
	Spain																			
	Switzerland																			
	The Netherlands																			[23]
	UK																			
	USA																			[64]
	WHO																			
LMWH	Australia							** &											**	[32]
	Belgium		*	**																[70]
	Canada																			
	France								&											[26]
	India																			[72]
	Italy																			[21]
	Norway																			[37]
	S. Arabia																			
	Spain																			[27]
	Switzerland						**													[28]
	The Netherlands																			[23]
	UK		**																	[81]
	USA					**														[64]
	WHO															**				[75]
	Color code			Strong recommendation for									Only in research settings							
				“Consider” or “can be offered”									Conditional recommendation against							
				“Under study/assessment” or unknown									Compassionate use							
				Conditional recommendation									Strong recommendation against							

In this table, there are two supplementary colors to WHO color code described in Table 1: light green for: “consider” or “can be offered” or “no absolute contraindication”, and pink for “compassionate use”. LMWH: low-molecular-weight heparin; IL: interleukin; IVIg: Intravenous Immunoglobulins; UK: United Kingdom; S. Arabia: Saudi Arabia; WHO: World Health Organization. † Medicine prescribed after individual evaluation by an infectious disease specialist (Norway). **Antibiotics’ prescriptions:** § Should be considered only if secondary bacterial infection suspected. **Antivirals’ prescriptions:** †† Side effects of remdesivir in pregnant women and consequences for pregnancy are unknown. If a decision is made to administer remdesivir in these patient categories, it is urged that patient data and outcomes be recorded through online data registration, so that more data are available for the pregnant women (the Netherlands). ††† Remdesivir in patients with non-severe COVID-19 at highest risk of hospitalization, and in patients with severe but not critical COVID-19 (WHO). **Corticosteroids’ prescriptions:** * For severe and critical COVID-19. α Hydrocortisone 150 mg daily if dexamethasone is not available (Belgium, India and Italy). β Methylprednisolone 32 mg daily if dexamethasone is not available (Belgium, India and Italy). χ Prednisolone 40 mg daily if dexamethasone is not available (Belgium and India). γ Methyl prednisolone boluses in case of acute respiratory distress and when inflammation predominates (Spain). δ Prednisolone 40 mg orally daily (UK). ε Hydrocortisone 80 mg twice daily (UK). ζ Hydrocortisone 100 mg twice daily, after dexamethasone if dexamethasone was indicated for the fetus (the Netherlands). λ Dexamethasone 12 mg daily for 2 days was recommended if severe COVID and additional indication of fetal lung maturation. If no additional fetal indication: prednisolone 40 mg daily or hydroxycortisone 50 mg × 3 daily IV was recommended for 7 to 10 days in case of severe respiratory failure (Norway). η Only for refractory shock COVID-19 patients: hydrocortisone 200mg daily IV (USA). φ Case by case decision for pregnant women pending additional results and with the respective specialists (Italy). **Immuno-suppressants and immunomodulators’ prescriptions:** ± Tocilizumab only in case of moderate severe disease with no response to corticosteroids or severe disease (the Netherlands). √ Convalescent plasma indicated only in immunocompromised patients (USA). **Anti-SARS-CoV-2 mABs’ prescription:** ≈ should be considered only in case of susceptible variants; all mABs currently authorized for COVID-19 are unlikely to be effective against emerging strains of SARS-CoV-2. In pregnancy, their use should be considered only if the potential benefit justifies the potential risk for the mother and the fetus considering all associated health factors (Italy). ∑ only in patients hospitalized with moderate to critical COVID-19 who are seronegative for SARS-CoV-2 antibodies. $ Sotrovimab (Australia). ∞ if SARS-CoV-2 antibody negative, decision depend on the variant, refer to the most up-to-date information from the Medicine and healthcare products regulatory agency (UK). **Anticoagulants’ prescriptions:** ** in case of hospitalization for COVID-19. & in case of individual risk factors for venous thromboembolism.

## 4. Discussion

### 4.1. Main Findings

This scoping review of the national guidelines on the medicines used to treat COVID-19 in pregnancy points out that many pharmacological options were investigated to treat COVID-19 in the first year of the pandemic, but their effectiveness after assessment remained limited or even absent, with the exception of corticosteroids in the case of severe or critical COVID-19 [2]. In the absence of evidence-based data in the early pandemic, pregnant women could in some cases benefit from drug repurposing, i.e., the reuse of drugs used in other medical indications but potentially beneficial in the COVID-19 patient. Lopinavir–ritonavir, oseltamivir and hydroxychloroquine were used in this context [46]. In 2020, the different national guidelines were initially discordant regarding the prescription of hydroxychloroquine, lopinavir–ritonavir and remdesivir. Comparisons between these different national guidelines published in March and April 2020 revealed a lack of uniformity and consistency, resulting in potentially challenging decisions for healthcare providers [57]. In 2021, with the results of the RCTs, the guidelines became much clearer and more consistent for these well-known medicines. From December 2021, anti-SARS-CoV-2 monoclonal antibodies could be prescribed under certain conditions in some countries but were no longer recommended by the Netherlands, USA and WHO at the end of 2022, as the predominant Omicron subvariants were not susceptible to these products. Finally, in 2022, the national guidelines were still not fully aligned regarding the indications for treatment with remdesivir, nirmatrelvir-lopinavir and tocilizumab.

### 4.2. Difficulties Encountered in Developing Guidelines to Treat COVID-19 in Pregnant Women

During the first wave of the pandemic, clinical recommendations issued in Asian, Australian, European, and North American countries were conflicting for many reasons.

First, pregnant women were not included in most RCTs, because of theoretical concerns on the potential impact of medicines on the fetus [82]. In April and July 2020, between 74% and 80% of COVID-19 treatment trials listed pregnancy as an exclusion criterion [10]. With regard to the largest clinical trials, pregnancy was not a specific exclusion criterion in the RECOVERY trials concerning the evaluation of lopinavir–ritonavir, corticosteroids, azithromycin, and tocilizumab [2,4,5,6]. The RECOVERY trials were allowed to include pregnant women conditional on the follow-up of maternal and fetal outcomes by the UK Obstetric Surveillance COVID-19 study. As of 21 April 2020, the international SOLIDARITY trial changed its inclusion criteria to allow pregnant women to participate [83]. The resulting paucity of safety and effectiveness data on COVID-19 treatment for this population was illustrated in the systematic review published by Giesbers et al. on pharmacological interventions to treat COVID-19 in pregnancy from December 2019 to December 2020. The authors did not find any RCT regarding COVID-19 treatment during pregnancy, but six studies (599 persons) were eligible. The number of pregnant women exposed to intervention was so small, that despite big denominators, it was not possible to conduct a meta-analysis and draw any conclusions about pharmacological interventions for treatment of pregnant persons with COVID-19 [84]. In light of the very limited use of drugs during pregnancy and the significant gaps in knowledge about the impact of medicines on perinatal outcomes, the authors encouraged researchers to include pregnant women in their trials and report the data on pregnant women separately [84].

Second, national guidelines on medicines used to treat COVID-19 may have been inconsistent because it was difficult to transpose recommendations from clinical trials conducted in nonpregnant adults to pregnant women. For most treatments, there were no data directly available in pregnancy, but also no rationale to suggest that the effect would differ significantly than the one observed in the general population. Interpretation of the studies may also have differed depending on the timing and intensity of the different waves of the pandemic locally, the national health care context, and the media coverage of the COVID-19 studies in different countries. It is important to note that the prices of antimalarial treatments and corticosteroids were affordable for many countries in the world. Antivirals and immunomodulators, on the other hand, are much more expensive, which could hamper their availability to all patients who need them, especially in low-income countries [85]. This cost-effectiveness has been taken into account in the Indian guidelines from 2020 [36]. The lack of available data specific to pregnant women is well illustrated by Table 3, in which some national guidelines were unable to give recommendations for many drugs. The lack of recommendations in pregnant women was also well illustrated by the COVID-19 task force in Switzerland that gathered a panel of 25 experts in adults and children, but no obstetrician [86].

Third, the publication process at the beginning of the outbreak was biased by the urgent need to obtain information about this new virus and its potential treatments, but also by the opportunity to publish the slightest piece of new information related to COVID-19 as quickly as possible. Indeed, there were 1′960, 2′987 and 3′241 published articles with the search query including the words “COVID*” AND “pregnancy” on PubMed in 2020, 2021 and 2022, respectively. It is more than all the articles ever published on cytomegalovirus infection during pregnancy. Moreover, between November 2019 and May 2020, the average time to review an article was significantly shorter for COVID-19 related publications (11.3 days for COVID-19 articles vs. 106.3 days for non-COVID-19 articles; *p* < 0.001). Moreover, thousands of COVID-19 publications have been released in preprint archives, before the peer review process [87]. The reconsideration, early in the pandemic, of the use of corticosteroids in fetal indication, which is a major revolution in obstetrics management, highlights very well the impact of this bustle on the clinical recommendations. Misinterpretation of some data in COVID-19 nonpregnant patients suggested that corticosteroids might be linked to increased morbidity and mortality. As a result, some authors reacted too hastily by recommending a change in standard practice regarding corticosteroid therapy and weighing maternal risk against fetal benefit [10].

Finally, another issue with the rapidly growing evidence was that recommendations were quicky out of date. This resulted in guidelines retraction during the first wave of the pandemic, because of new information released at such an incredible rate that the published guidelines could not remain up to date for more than a few months. As an example, the Expert Committee of the Society of Obstetricians and Gynaecologists of Canada (SOGC) decided to remove their guidelines published in March 2020 because they were out of date [88]. The authors formally requested that one visits the website of the SOGC for an online version of the Committee Opinion which is regularly updated as new evidence emerges.

### 4.3. Response through Constantly Updated Online Treatment Guidelines

This overview of national guidelines mostly published by the obstetricians’ societies on their official websites illustrates a need to provide national “rapid response” recommendations to assist health care providers in their national language at the beginning of the pandemic, despite a lack of scientific evidence. The objectives of the national guidelines were to be easily accessible by health care providers in maternity wards, and to be adapted to local health environments [89].

Thus, the national guidelines development process needs to be dynamic and rapid, which means that the guidance is updated quickly as new evidence is released. With thousands of papers being published each week on COVID-19 epidemiology and treatments, the guidelines are of utmost importance to synthetize and clarify the treatment guidelines for clinicians and consumers [90]. Therefore, national guidelines have been constantly being updated and expended in 2020–2022, with many versions and updates, preferentially in the form of living or interim guidelines on the national websites (Table 2). As a result, the living guidelines on COVID-19 with specific considerations for pregnancy reported by NIH guidelines have been updated 62 times in three years [64]. In this regard, most USA guidelines refer to those of the NIH concerning medication, which are regularly updated as soon as new information on the management of COVID-19 becomes available, and also provides specific sections for pregnant women [20,35].

### 4.4. Strength

The strength of this historical review is that our working group is composed of obstetricians–gynecologists, pharmacologists, pharmacists, and epidemiologists specialized in perinatal health, who reviewed all versions of their national guidelines published in their national language and merged them into this review [91]. This study is of great importance for the interpretation of studies on COVID-19 in pregnancy conducted in 2020–2022, because pregnant women were treated inconsistently across different study periods and countries. This description of the evolution of COVID treatment over time makes it possible to contextualize the studies published during the first three years of the pandemic and to recall how much physicians lacked reliable references at its beginning.

### 4.5. Limitations

The main limitation of the review is that we focused on 13 countries, which are mainly high-income level countries. Thus, our findings are rather generalizable to countries with similar health care structures, whereas the impact of COVID-19 in pregnant individuals has been shown to differ between high-income and low- and middle-income countries [92]. Secondly, our search was restricted to the websites of the national societies and PubMed, whereas there are likely alternative ways to disseminate guidelines through apps and social media [93]. Moreover, the Spanish Society of Gynecologists and Obstetricians’ (SEGO) guidelines were published in May and June 2020 and do not reflect the reality of the treatments that have been administered to Spanish pregnant women. The protocol from BCNatal, a tertiary maternal–fetal center in Barcelona has been updated 17 times up to the end of 2022 [94]. Finally, we compared guidelines that did not use the same methodology (expert opinion vs. living guideline), which may have contributed to different conclusions linked to the nature of the literature review.

The management of COVID-19 in pregnancy requires a multidisciplinary approach, with a clinical assessment considering the risk of deterioration, hypoxia, need for ventilatory support and decisions of ongoing care vs. induced delivery. Collaboration between specialists (obstetricians-gynecologists, midwives, anesthetists, pediatricians, specialists in infectious diseases, pharmacists, pharmacologists, pharmacoepidemiologists and hematologists) is of utmost importance to harmonize national recommendations and have a unique reference. This multidisciplinary collaboration has been leveraged in many reported national guidelines. Our scoping review of the medicines recommended by professional societies to treat COVID-19 in pregnancy in the three first years of the pandemic depicts how health workers have adapted to an emergent virus, growing scientific evidence and new strains with potential resistance to medication. Researchers responded swiftly to this pandemic, thanks to international collaborations for conducting clinical trials, but unfortunately neglected to include pregnant women. Yet, since the beginning of the pandemic, experts in maternal fetal medicine have recommended and urged the scientific community and professional societies that, without clear justification for exclusion, pregnant women should be given the opportunity to be included in clinical trials for COVID-19 based on the concepts of justice, equity, autonomy, and informed consent. This is a lesson to be learned and to be carried out in case of a potential future pandemic [10,82,95].

In addition, the authors believe that vaccinations are still the safest way to protect against severe COVID-19 infection in pregnancy, although there are challenges for universal vaccination, including vaccination hesitancy and availability in developing nations [96].

## 5. Conclusions

Since March 2020, our knowledge of COVID-19 and treatment modalities has evolved significantly. A comparison between the different guidelines published in spring 2020 revealed a lack of uniformity and consistency, resulting in potentially challenging decisions for healthcare providers. Recommendations became much more consistent in 2021 for corticosteroids and well-known antivirals. However, in 2022, national guidelines still differed for remdesivir and nirmatrelvir–ritonavir between countries. Recommendations on anti-SARS-CoV-2 monoclonal antibodies evolved between December 2021 and December 2022, as the predominant Omicron subvariants were not susceptible to these products.

The national guidelines to treat pregnant women with COVID-19 produced by the National COVID-19 Clinical Evidence Taskforce in Australia, the RCOG in the UK and the NIH in the USA have been, and continue to be, essential resources for doctors, nurses and midwives who provide acute and primary maternity care services. They are developed using highly trusted methods and are endorsed by national health professional organizations. As of April 2023, the section devoted to pregnancy in the NIH guidelines recommended against withholding COVID-19 treatments or vaccination from pregnant individuals specifically because of pregnancy. In general, the therapeutic management of pregnant patients with COVID-19 should be the same as for nonpregnant patients, with a few exceptions, such as a recommendation against the use of molnupiravir for the treatment of COVID-19 in pregnant patients, unless there are no other options and therapy is clearly indicated. A prophylactic dose of anticoagulation is recommended for pregnant patients who are hospitalized for manifestations of COVID-19, unless a contraindication exists [97].

## 6. Future Directions

Multidisciplinary and international collaborations are paramount in the context of a pandemic with a new pathogen. However, the inclusion of pregnant women in COVID-19 clinical trials and the publication of pregnancy-specific findings in an independent manner would allow the evaluation of effective therapies that might improve maternal, pregnancy, and neonatal outcomes, and avoid the delay of developing treatment recommendations for pregnant women. Regulatory authorities, the pharmaceutical industry and academia have a joint responsibility to ensure that pregnant women are included in clinical trials and pharmacovigilance studies in future pandemics.

## Figures and Tables

**Table 1:** Recommendation grading derived from World Health Organization and Australian living guidelines.

Color	Grading	Color Code Meaning in WHO Guidelines	Color Code Meaning in Australian Guidelines
	Strong recommendation for	It is better to prescribe based on strong evidence. The benefits outweigh the harms for almost everyone. All or nearly all informed patients would likely want this option.	There is high-certainty evidence showing that the overall benefits of the intervention are clearly greater than the disadvantages. This means that all, or nearly all, patients will want the recommended intervention.
	Conditional recommendation for	The medication is indicated in some categories of patients, such as patients with comorbidities, or patients with seronegative status for COVID-19. Benefits outweigh harms for the majority, but not for everyone. The majority of patients would likely want this option.	A conditional recommendation is given when it is considered that the benefits of the intervention are greater than the disadvantages, or the available evidence cannot rule out a significant benefit of the intervention while assessing that the adverse effects are few or absent.
	“under study” or “under assessment” or unknown	The national societies are not able to conclude at the time of their recommendations because of insufficient evidence in the literature.	
	Only in research settings	The experts recommend not to use the medication, except in the context of a clinical trial.	The panel recommends that the intervention should only be considered in a randomized clinical trial with appropriate ethical approval. In any other circumstance, the intervention is not recommended.
	Conditional recommendation against	It is not recommended to prescribe a drug in some categories of patients, such as patients who do not require oxygen therapy, because it has been shown to be ineffective. A majority would likely decline the intervention.	A conditional recommendation is given against the intervention when it is judged that the disadvantages of the intervention are greater than the benefits, but where this is not substantiated by strong evidence. This recommendation is also used where there is strong evidence of both beneficial and harmful effects, but where the balance between them is difficult to determine.
	Strong recommendation against	It is not recommended to prescribe a drug because it has been shown to be ineffective, or even potentially harmful. All or nearly all would likely decline the intervention.	There is high-certainty evidence showing that the overall disadvantages of the intervention are clearly greater than the benefits. A strong recommendation is also used when the examination of the evidence shows that an intervention is not safe.

WHO: World Health Organization. In the WHO and Australian guidelines, “only in research setting” and “conditional recommendation against” have the same color. It has been slightly changed for better understanding.

**Table 2:** References of national guidelines on treatments in pregnant women with COVID-19: timeline from March 2020 to December 2022 in 13 countries and World Health Organization.

	Guidelines in Pregnant Women or General Population with Specific Section for Pregnancy	Date of First and Latest Version until 31 December 2022	Number of Versions	Guidelines Methodology (Number of References)	Other National Societies (Involved and/or Collaborating)
Australia	National COVID-19 Clinical Evidence Taskforce [22]	14 May 2020 19 December 2022	9	Living guideline, GRADE methodology (29 ref.)	RANZCOG: Royal Australian and New Zealand College of OBGYNs ACM: Australian College of Midwives
Belgium	AFMPS (Agence fédérale des Médicaments et Produits de Santé)–FAGG [24]	17 April 2020 December 2022	33	Living guideline (256 ref.)	SBIMC: Société Belge d’Infectiologie et de Microbiologie Clinique Sciensano: National Institute of Public Health CBiP: Centre Belge d’information Pharmacothérapeutique
Canada	SOGC (Society of Obstetricians and Gynecologists of Canada) [18]	17 March 2002 15 February 2021	4	Expert opinion (70 ref.)	-
France	CNGOF (Collège National des Gynécologues-Obstétriciens français) [26]	19 March 2002 5 October 2020	2	Expert opinion (98 ref.)	DGS: General Direction of Health HCSP: French High Council for Public Health HAS: French National Authority for Health CARO: Club of Anesthetists in Obstetrics
India	FOGSI (Federation of Obstetric and Gynaecological Societies of India) [36]	28 March 2020 16 June 2021	3	CPG, GRADE approach (168 ref.)	NNF: National Neonatology Forum of India IAP: Indian Academy of Pediatrics ICMR: National Institute for Research in Reproductive Health
Italy	AIFA (Italian Medicines Agency) [21]	Information sheets on individual drugs			-
Norway	Norsk Gynekologisk Forening (Norwegian Society of Gynecology and Obstetrics) [37]	24 March 2020 22 March 2022	7	Expert opinion (85 ref.)	OBGYNs, infectious disease specialists, microbiologists, epidemiologists and pharmacologists involved
Saudi Arabia	Saudi Society of Maternal-Fetal Medicine [34]	1 August 2020 -	1	Expert opinion (6 ref.)	Saudi Ministry of Health [17]
Spain	SEGO (Sociedad Española de Ginecología y Obstetricia) [27]	13 May 2020 17 June 2020	2	Expert opinion (90 ref.)	SENEO: Sociedad Española de Neonatología SEDAR: Sociedad Española de Anestesiología, Reanimación y Terapéutica del Dolor SETH: Sociedad Española de Trombosis y Hemostasia SEEN: Sociedad Española de Enfermería Neonatal AEM: Asociación Española de Matronas FAME: Federación de Asociaciones de Matronas de España IHAN: Iniciativa para la Humanización de la Asistencia al Nacimiento y Lactancia CGCOM: Consejo General de Colegios Oficiales de Médicos CGE: Consejo General de Enfermería
Switzerland	SSGO (Swiss Society in Gynecology & Obstetrics) [28]	5 August 2020 7 January 2022	2	Expert opinion (28 ref.)	-
the Netherlands	FMS (Federatie Medisch Specialisten) and NVOG (Nederlandse Vereniging voor Obstetrie en Gynaecologie) [38]	1 June 2020 8 November 2022	7	Expert opinion (40 ref.)	RIVM: Rijksinstituur voor Volksgezondheid en Milieu SWAB: Stichting Werkgroep AntibioticaBeleid KNOV: Koninklijke Nederlandse Organisatie van Verloskundigen NVA: Nederlandse Vereniging voor Anesthesiologie NVK: Nederlandse Vereniging voor Kindergeneeskunde NVMM: Nederlandse Vereniging voor Medische Microbiologie K&Z: Stichting Kind en Ziekenhuis NVZ: Nederlandse Vereniging van Ziekenhuizen
UK	RCOG (Royal College of Obstetricians and Gynaecologists) and Royal College of Midwives [39]	9 March 2020 15 December 2022	16	Expert opinion (199 ref.)	Royal College of Paediatrics and Child health, Royal College of Anaesthetists NICE: National Institute for Health and Care Excellence
USA	SMFM (Society for Maternal Fetal Medicine) [20] *	17 March 2020 21 June 2022	10	Expert opinion (48 ref.)	ACOG: American College of OBGYN
	NIH (National Institutes of Health) [40]	21 April 2020 28 December 2022	62	Living guideline (around 30 ref. per section)	CDC: Center for disease Control and Prevention
“World”	WHO (World Health Organization) [41]	2 September 2020 14 July 2022	10	Living guideline, GRADE methodology (168 ref.)	-

CPG: Clinical Practice Guidelines; GRADE: Grading of Recommendations Assessment, Development and Evaluation; OBGYN: Obstetricians and Gynecologists; * SMFM, ACOG and CDC support the NIH COVID-19 treatment guideline.

## Data Availability

The data presented in this study were available online and accessed on 15 June 2023.
